# The Way Ahead: Life After COVID-19

**DOI:** 10.14797/mdcvj.1056

**Published:** 2021-12-15

**Authors:** Mouaz H. Al-Mallah

**Affiliations:** 1 Houston Methodist DeBakey Heart & Vascular Center, Houston, TX, US

**Keywords:** COVID-19, coronavirus, cardiovascular disease, vaccine

## Abstract

Much has changed in the 2 years since the start of the coronavirus disease 19 (COVID-19) pandemic. The need for social distancing catalyzed the digitization of healthcare delivery and medical education—from telemedicine and virtual conferences to online residency/fellowship interviews. Vaccine development, particularly in the field of mRNA technology, led to widespread availability of safe and effective vaccines. With improved survival from acute infection, the healthcare system is dealing with the ever-growing cohort of patients with lingering symptoms. In addition, social media platforms have fueled a plethora of misinformation campaigns that have adversely affected prevention and control measures. In this review, we examine how COVID-19 has reshaped the healthcare system, and gauge its potential effects on life after the pandemic.

## Introduction

**Figure 1:**
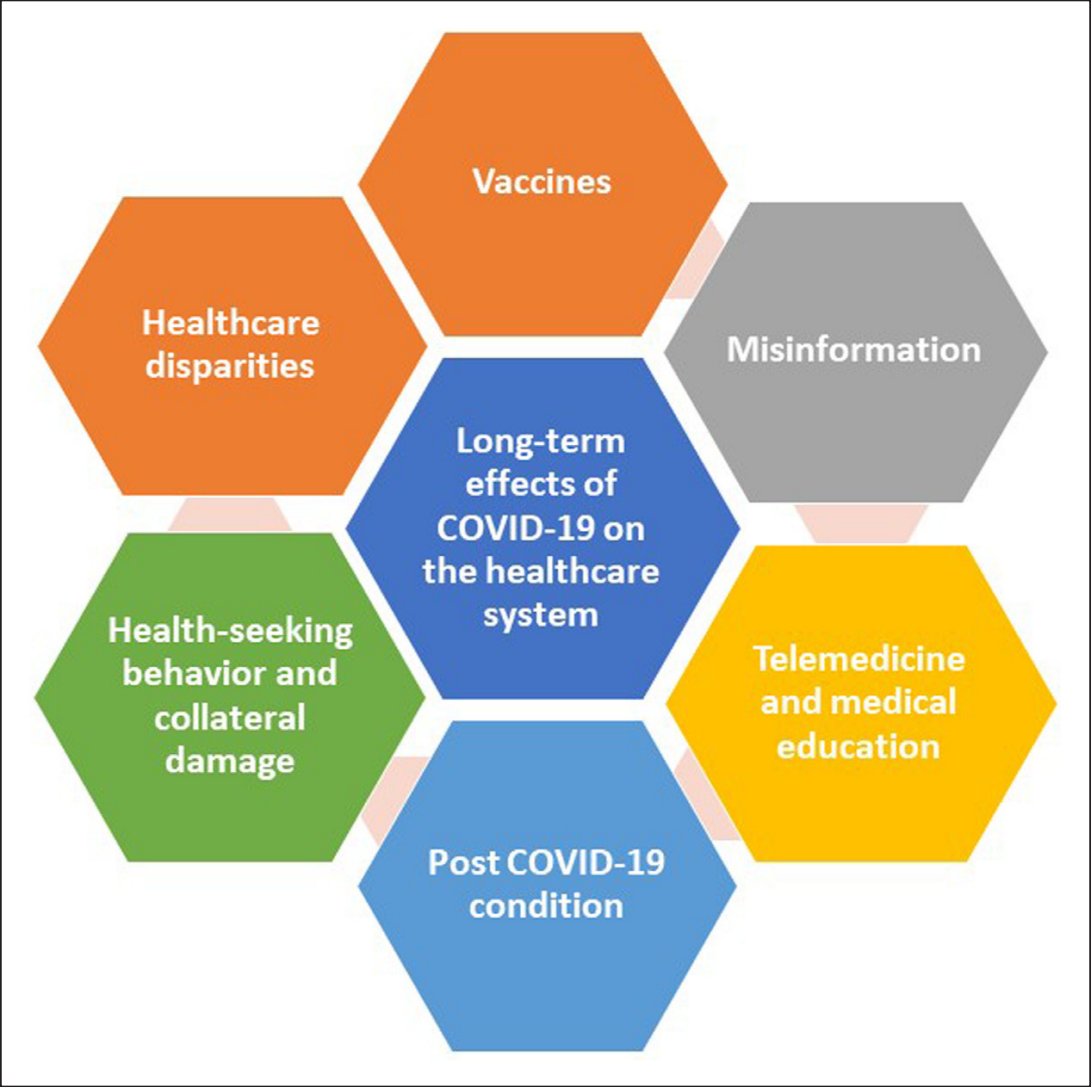
The long-term effects of the coronavirus disease 19 (COVID-19) pandemic on the healthcare system.

In December 2021, after many months of living with the COVID-19 pandemic, the world is still looking for a way out of this healthcare crisis. As of this writing, more than 250 million people globally have been infected with SARS-CoV-2, the virus that causes coronavirus disease 19 (COVID-19), and nearly 5 million individuals lost their lives battling the complications of severe acute respiratory syndromes.^1^ Many communities experienced multiple surges of the virus, with changes in normal life and restrictions to daily activities. The intensification of vaccination efforts brought about hope for a possible end to the pandemic. However, the continued emergence of variant strains and vaccine hesitancy have been persistent challenges in the US and globally. In this article, we review the long-term effect of COVID-19 on healthcare systems and envision the future of life after the pandemic (***Figure 1***).

## Vaccines

Since the beginning of the pandemic, there have been accelerated efforts to sequence the genetic material of the virus and build effective vaccines that decrease the risk of infection, hospitalization, and mortality.^2^ At the time of this writing, more than 10 vaccines have been approved by local healthcare authorities in different parts of the world.^3^ The pandemic has also driven innovation in the novel field of messenger ribonucleic acid (mRNA) vaccines. The US Food and Drug Administration (FDA) has approved the use of the Pfizer-BioNTech mRNA vaccine and given emergency use authorization to Moderna.^4^ The mRNA vaccines have shown excellent efficacy against many of the strains, including the beta and delta strains.

More recently, booster doses have been approved by the FDA for individuals aged 65 years and older as well as individuals with comorbidities, in long-term care facilities, or at increased risk for COVID-19 exposure and transmission due to occupational or institutional settings.^5^ Furthermore, the FDA has also given emergency use authorization for the Pfizer-BioNTech vaccine in individuals aged 12 to 17 years and, as of October 29, in children aged 5 to 11 years.

Although the fast-tracked vaccine production time led some skeptics to hypothesize safety concerns, the rate of adverse events has been very low. One complication that gained significant attention is myocarditis.^6,7,8^ Emerging data have shown that young men are the most commonly affected demographic. Furthermore, the risk was elevated in the setting of a recent COVID-19 illness and after the second dose of the vaccine.^6,7^ Although the rate of myocarditis is low and the majority of patients recover, the risk of recurrence in patients who developed myocarditis with the first dose or in patients with recent myocarditis is unclear. Similarly, the rate of recurrence after the second or booster doses also is unclear.

## Vaccine Mandates

Multiple state and federal governments have issued vaccine mandates, and they have become a highly contested political issue in the United States. The Biden administration issued an executive order on September 9, 2021, requiring all federal employees to vaccinate.^9^ Some state and local governments have also followed.^10^

Multiple US healthcare systems have also issued COVID-19 vaccine mandates for employees. On March 31, 2021, Houston Methodist became the first healthcare system to mandate the vaccine for employees, and a wave of other healthcare systems followed suit.^11^ As of this writing, more than 2,500 hospitals or health systems have followed Houston Methodist and mandated vaccines for their clinical and nonclinical staff.^12^

## Combating Misinformation

Since the beginning of the pandemic, misinformation has spread throughout the Internet and on social media platforms.^13^ People have questioned the existence of the virus, the strain on healthcare systems, and the benefit of masks as well as emphasized the benefits of unproven therapies, many of which were useless and even harmful.^14^ Political agendas have also played into the misinformation campaigns. Studies have shown that these misinformation campaigns have had measurable effects on the intent to vaccinate and created widespread fear and panic, ultimately contributing to the reduced number of people willing to vaccinate.^13,15,16^ Tackling this will require concerted efforts by the government and private sector, particularly social media companies, to implement evidence-based communication strategies.^17^ Individuals should also assume responsibility in seeking out accurate, evidence-based information for their own consumption.

## Telemedicine

As many states and cities implemented measures to reduce transmission, telehealth emerged as the ideal tool to continue patient care while protecting the health of both patients and providers. Many patients preferred this option, especially when hospitals were dealing with record numbers of COVID-19 infections. In 2020, telemedicine was the main means by which ambulatory care was provided, accounting for 10% to 20% of visits when virus transmissibility was low and as high as 80% of visits during the surges.^18^

Accordingly, the US Department of Health and Human Services relaxed enforcement of software-based Health Insurance Portability and Accountability Act violations, the Centers for Medicaid and Medicare Services provided waivers for telehealth reimbursements, and, in many instances, commercial insurances provided the same either directly or through mandates provided by local state governments.^19,20^ The removal of regulatory and reimbursement barriers led to a dramatic increase in the use of telehealth, with some institutions reporting multifold increase in telehealth visits.^21^

The pandemic also served as a catalyst for innovation in the software and hardware necessary for telemedicine.^22^ For example, important tools were developed to enable secure connections with physicians and allow remote vital sign and weight monitoring.^23,24^ Unfortunately, not all have equally benefitted from the expanded use of telehealth. Data indicate that minorities and disadvantaged groups often lack access to telehealth-based care.^25^ Although the positive response and uptake by physicians and patients indicates the likelihood of telemedicine continuing past the pandemic, it remains to be seen whether the regulatory and reimbursement aspects will continue.

## Post Covid-19 Condition

There is a growing body of evidence that some patients have prolonged recovery and/or residual symptoms after acute infection with COVID-19. The World Health Organization has defined this as “post COVID-19 condition.” Common presentation includes shortness of breath, palpitation, anxiety, and depression lingering for several months after acute infection.^26,27^ Recent data also suggests that post COVID-19 condition might not be limited to somatic symptoms, with studies showing a 7-fold increased risk of developing depression and mental health issues.^28^

Although the cause of these symptoms is not clear, one possible link that partly explains the prolonged shortness of breath experienced by some patients is COVID-19–associated myocarditis and the associated microvascular dysfunction.^26^ As the pandemic continues and therapeutics improve survival from acute infection, the number of patients reporting post COVID-19 condition is predicted to grow. Several medical centers have already established clinics to better coordinate care and conduct research on the long-term impact and treatment of COVID-19.^29^

## Collateral Damage

Many patients delayed regular and preventive care during the pandemic due to fear of contracting COVID-19.^30,31^ Such change in health-seeking behavior also extended to emergency conditions, with studies showing how some patients did not seek care for new onset chest pain.^32^ Indirect indicators of this are the reduced rates of cardiovascular testing globally and within the United States^33,34^ and the increased rate of myocardial infarctions and other emergencies seen on the trailing end of COVID-19–infection surges.^32^ There has also been an increase in late complications of myocardial infarction such as ventricular septal rupture, a rare occurrence in the prepandemic reperfusion era and one partly explained by delayed care and ignored early warning signs.^35^

## Disparities in Healthcare

The pandemic exposed significant disparities in healthcare delivery, particularly among minorities. They were more likely to be affected by misinformation campaigns and less likely to accept research supporting clinical therapies and vaccines. Understanding the disparities and identifying measures to bridge the gap will be an important area of research for policy.

Globally, the pandemic also exposed significant inequities regarding vaccine access. While many developed countries were able to reach vaccination rates as high as 70%, rates in low-to-middle-income countries have remained low.^35^ As the delta variant has clearly shown, no one is safe until everyone is safe. To this end, the World Health Organization and the COVAX (COVID-19 Vaccines Global Access) alliance have been a vital source of affordable vaccines.^36^

## Changes to Medical Education

The pandemic resulted in significant changes to both graduate and continued medical education. Much like patient-physician encounters, postgraduate training programs limited large face-to-face gatherings and transitioned all teaching to online platforms.^37^ Residency and fellowship recruitment interviews also shifted to online settings. Lastly, there has been an exponential increase in the number of continued medical education offerings, with many societal meetings and conferences transitioning to online or hybrid formats.^38^

The medical community has, for the most part, been very receptive to these changes, and it has afforded unforeseen advantages to trainees. Residency and fellowship applicants no longer need to bear the logistic and financial burden of in-person interviews. More importantly, virtual meetings and conferences have significantly increased audiences and, by extension, enabled the wider dissemination of medical knowledge.

## Conclusion

The COVID-19 pandemic has dramatically changed clinical practice, medical education, and research. Beyond the immediate increase in morbidity and mortality, the healthcare system is having to deal with a growing cohort of patients with lingering symptoms. Misinformation, vaccine hesitancy, and vaccine inequity will be continuing challenges to attaining herd immunity. Clinicians, educators, and healthcare administrators will also have to determine how best to leverage the transition to virtual platforms. Lastly, healthcare leaders and policy makers will have to help the country and world chart a course through the end of the pandemic.

## Key Points

- The coronavirus disease 19 (COVID-19) pandemic has dramatically changed clinical practice, medical education, and research.
- It has brought about new challenges for the healthcare system, such as how best to combat misinformation, address the disproportionate impact on minorities and marginalized groups, and treat the ever-growing population of patients with lingering “long COVID” symptoms.
- The pandemic has also catalyzed much needed change in vaccine development, telemedicine, and medical education.
- Addressing these challenges and charting a way forward will require the concerted effort of clinicians, healthcare leaders, and policy makers.
